# Robust, Fluorine-Free Superhydrophobic Films on Glass via Epoxysilane Pretreatment

**DOI:** 10.1021/acs.langmuir.4c02630

**Published:** 2025-01-16

**Authors:** Fang Chen, Julie Jalila Kalmoni, Shuhui Li, Claire J Carmalt

**Affiliations:** Materials Chemistry Centre, Department of Chemistry, University College London, 20 Gordon Street, London WC1H 0AJ, U.K.

## Abstract

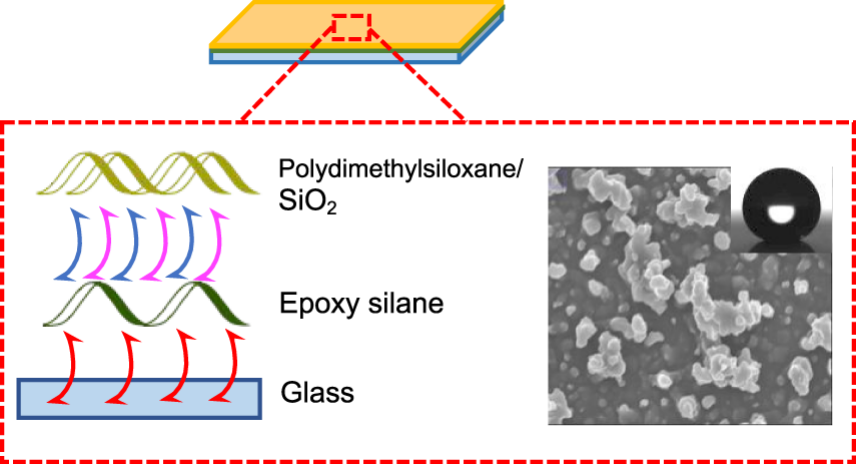

Durable and fluorine-free superhydrophobic films were fabricated by a simple two-step process involving the pretreatment of glass substrates with an epoxysilane, which acted as an adhesive. The next step involved the aerosol-assisted chemical vapor deposition of a simple mixture of polydimethylsiloxane (PDMS) and SiO_2_ nanoparticles (NPs). Various parameters were studied, such as deposition time as well as PDMS and SiO_2_ loadings. The optimum film generated was with a 1:1 loading of PDMS and SiO_2_, deposited at 360 °C for 40 min. The resultant film demonstrated excellent water repellency with a water contact angle of 165 ± 3° and a sliding angle of 2°. The epoxysilane underlayer provided the adhesion between the film and substrate. The films maintained superhydrophobicity and durability after being exposed to solvents such as diethyl ether, toluene, and ethanol for up to 5 h, 400 tape peel cycles, UV exposure, and heat exposure at 400 °C. The robustness results indicated enhanced durability relative to the superhydrophobic film without the epoxysilane underlayer.

## Introduction

Superhydrophobic (SH) surfaces have a water contact angle (WCA) > 150°, sliding angle (SA) < 10°, and contact angle hysteresis (CAH) < 10°.^1,2^ Due to their water repellency and self-cleaning abilities, these coatings have potential applications in oil–water separation, anti-icing, and antifogging.^3−8^ However, the creation of SH films require micro/nano-scale roughness and low surface energy reagents such as fluoroalkysilanes, which are known to be toxic for the environment.^9−11^ Therefore, there has been a shift towards employing fluorine-free polymers such as polydimethylsiloxane (PDMS).^12,13^ PDMS has a low surface energy of 19.8 mJ/m^2^ (fluoroalkylsilanes have surface energies between 17 and 20 mJ/m^2^) and a good film-forming ability.^14^ In addition, PDMS is an inexpensive and environmentally friendly material.

SH materials are notorious for their poor durability and adhesion to substrates, limiting their current applications. In addition their delicate nano/microstructures are susceptible to damage, affecting the water-repellency of the films. Hence, in addition to using an alternative low surface energy reagent, there is a pressing need to improve the robustness and adhesion of the coatings to substrates. For example, Wang et al. devised a morphology where the microstructures would act as an “armor”, protecting the delicate nanostructures from external forces and conditions, providing resistance to acids, bases, and high temperatures.^15^ Conceptually, this idea worked, but it is currently difficult to scale up for industrial applications. Hence, there has been interest in facile and scalable methods for fabricating robust SH materials. Binders and adhesives have been used widely due to their ability to improve and generate inter- and intramolecular forces, enhancing the chemical interactions between the substrate and coating. Polizos et al. fabricated a highly durable film by treating the glass substrate with a polymer binder (Cerakote) prior to spray-coating a mixture of the polymer binder and SiO_2_ nanoparticles (NPs).^16^ Xue et al. employed aluminum phosphate, which acted as an adhesive between the polystyrene NPs and cotton fibers, generating electrostatic interactions and hydrogen bonds. This rough composite was dip-coated with PDMS, a low surface energy reagent, attaining superhydrophobicity.^17^

Alternative adhesives that have been studied include epoxy resins (ER), which are polymers consisting of reactive epoxide groups. As a result, the epoxide groups can undergo a ring-opening reaction, forming hydrogen bonds with the O-containing bonds (e.g., O–Si) and the substrate’s hydroxyl groups, improving the film’s overall durability.^18^ For example, Guo et al. spray coated a mixture of ER, PDMS, and SiO_2_ NPs onto a glass substrate pretreated with ER.^19^ Overall, the film had a WCA of 163° and excellent durability, demonstrating its ability to tolerate sandpaper abrasion and water impingement tests. Zhuang et al. reported a well-adhered SH material with resistance to chemical, UV, and mechanical durability tests. Three layers of ER were deposited onto glass substrates via aerosol-assisted chemical vapor deposition (AACVD) at dynamic temperatures, followed by low surface energy treatment by immersing the coated substrate in a PDMS solution.^20^ Apart from ER improving a material’s overall durability, its resultant films can potentially self-heal, whereby a film’s superhydrophobic nature can be restored after damage under moderate external conditions such as UV, pH, external heat, or even at room temperature.^21^

Epoxysilanes (ES), another potential adhesive, have been studied but not as extensively as ER. Reports have shown that ES can enhance the adhesion and mechanical durability of films as they are good binders.^22,23^ ES can adhere to conventional materials, such as metals and glass, as well as organic polymers via a ring-opening reaction of their epoxide group. To further improve the adhesion of the ES to the substrate, the latter can be pretreated by methods such as chemical and/or plasma cleaning to enhance the quantity of surface hydroxyl groups.^24^ More importantly, they are inexpensive and environmentally safe.

SH films can be deposited via various deposition techniques, such as spray coating, sol–gel, hydrothermal methods, dip-coating, and spin-coating.^25−28^ However, most fabrication techniques need specific conditions or are inefficient, limiting large-scale implementation.^29^ Thus, there is a pressing need for a facile, scalable method. AACVD is a simple process with potential for industrialintegration; AACVD is a form of CVD that has been extensively used to deposit SH materials.^20,30−32^ An ultrasonic humidifier is used to generate aerosols of various sizes, which after passing through to the heated reactor, results in film deposition on the substrate, contributing to the overall roughness—a requirement for SH films.^33^ Additional benefits of AACVD are that precursors must be soluble in the carrier solvent rather than volatile in order to generate the aerosols.^34,35^ All of the mentioned benefits give us access to more precursors that could not have been used for deposition of thin films via conventional CVD, facilitating the fabrication process and reducing the total cost.

Herein, a simple, new route to fabricate fluorine-free superhydrophobic films has been devised, which involves depositing a mixture of PDMS and SiO_2_ NPs onto a glass substrate with an ES base coating via AACVD. In addition to including the traditional PDMS and silica NPs to deposit a superhydrophobic film, a novel technique combining brushing of an ES and AACVD has been used to fabricate durable superhydrophobic films. The use of an ES underlayer in addition to the superhydrophobic film has enhanced the durability of the superhydrophobic film. The ES used was 3-glycidyloxypropyltrimethoxysilane (GLYMO), an organic silane consisting of an Si atom attached to a single epoxide group and three methoxy groups. The ES underlayer worked as an adhesive between the film and glass substrate. The SiO_2_ NPs generated the roughness and transparency, and PDMS was used to lower the surface energy and contribute to the roughness by sticking the particles together due to its viscous nature. The mechanical robustness of the coatings was improved significantly by applying an ES underlayer for our SH film to grow on. The precursor composition (namely, PDMS and SiO_2_ loadings) and deposition conditions (temperatures and durations) were varied to determine the optimum parameters for producing well-adhered superhydrophobic films with good transparency.

## Experimental Section

Vinyl-terminated polydimethylsiloxane (PDMS), specifically Sylgard-184 Silicone Elastomer Base and its corresponding curing agent, were bought from Dow Corning. Aerosil OX50 fumed SiO_2_ NPs (diameter = 40 nm) were purchased from Lawrence Industries. The epoxysilane, namely, 3-glycidyloxypropyltrimethoxysilane (GLYMO), ethyl acetate (laboratory grade), and methanol (laboratory grade), were purchased from Sigma-Aldrich. All of the reagents were used as purchased. Pilkington NSG supplied barrier-coated float glass, which was cut into pieces of 15 × 4 × 0.3 cm, needed for AACVD.

A Henniker plasma cleaner (HPT-100) was used to plasma-treat the surface of the glass substrate prior to film deposition. The following parameters were used: a gas flow of 10 sccm and a duration of 5 min.

### Synthesis of ES/PDMS/SiO_2_ Films

**Figure 1:**
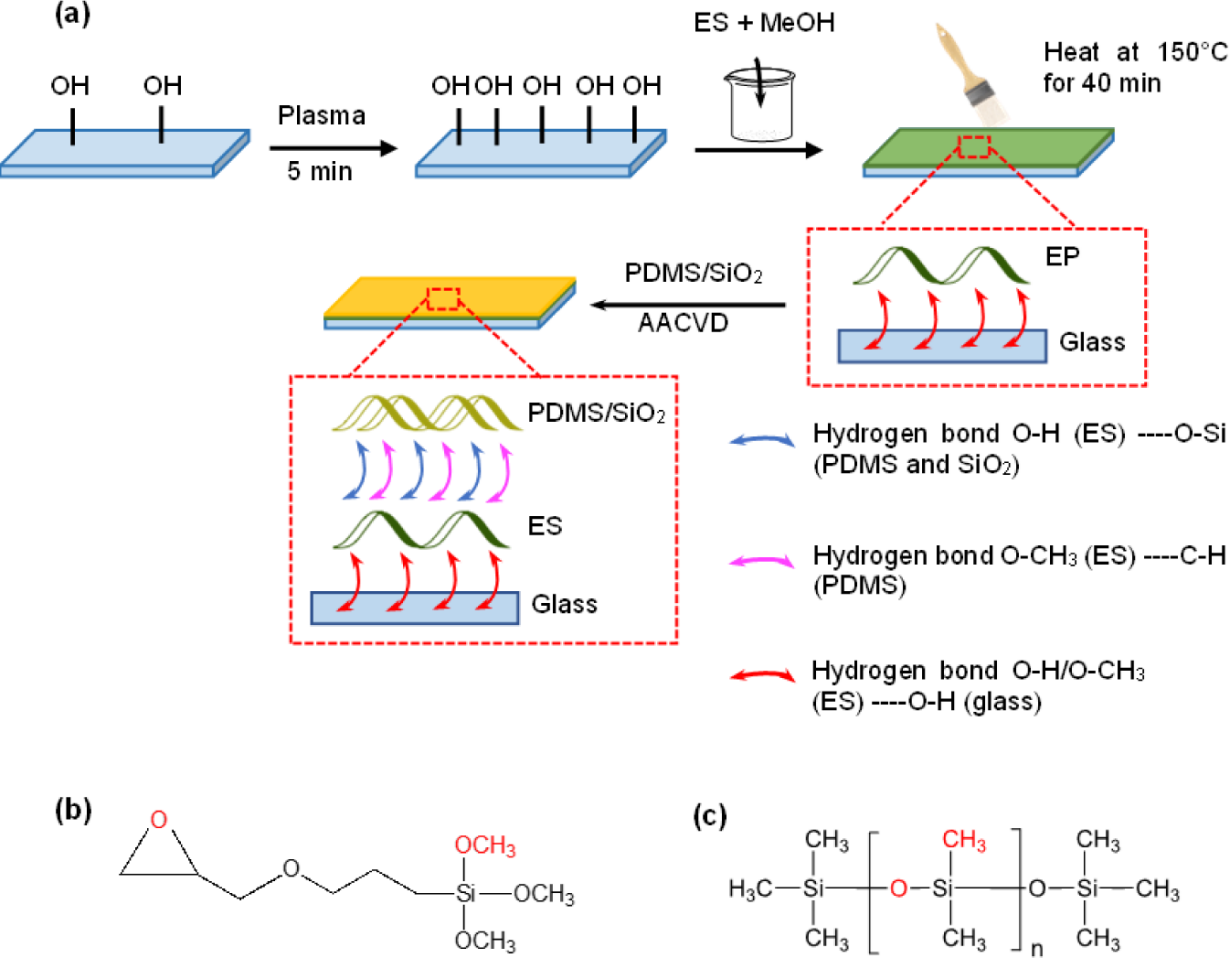
(a) Schematic describing the synthesis process of the ES/PDMS/SiO_2_ films. Chemical structures of (b) GLYMO and (c) PDMS.

The barrier-coated glass substrate was cleaned with acetone, soap, water, and isopropanol before it was plasma cleaned with a gas flow of 10 sccm for 5 min. GLYMO (2 mL) was added to methanol (5 mL) and stirred for 15 min. Subsequently, the solution was brushed uniformly onto the plasma-treated substrate with a brush and then left in an oven at 150 °C for 40 min to evaporate the methanol and encourage a reaction between the ES and glass substrate. This formed the ES-pretreated glass substrate. Sylgard-184 (0.6 g) and its curing agent (0.06 g) were dissolved in ethyl acetate (50 mL) for 5 min before OX50 silica NPs (0.6 g) were added to the solution and mixed for an extra 25 min, forming the ES/PDMS/SiO_2_ precursor mixture. The experimental method is schematically represented in Figure 1. Subsequently, the ES-pretreated glass substrate was inserted into the AACVD rig as a top plate in a bottom-down heating configuration, as described previously.^36^ Thus, the film precursor mixture was deposited onto this glass top-plate (substrate). The graphite heating block used a Whatman cartridge heater regulated by a Pt–Rh cartridge heater. This setup was enclosed in a cylindrical quartz tube. The precursor mixture was deposited onto the top plate substrate via thermophoretic effects. At 360 °C, the desired deposition temperature, a piezoelectric ultrasonic humidifier was used to generate an aerosol which, with the N_2_ carrier gas (1 L min^–1^), traveled through to the heated chamber for 40 min. After deposition, the film was cooled to room temperature under N_2_.

Various parameters were studied such as the loadings of xPDMS (where *x* = 0, 0.2, 0.3, 0.4, 0.6, and 0.8 g), loadings of *x*SiO_2_ (where *x* = 0, 0.2, 0.4, 0.6, and 0.8 g), and lengths of deposition (15, 20, 25, 30, 35, and 40 min). A summary of films fabricated under different conditions is shown in Table 1.

**Table 1:** Summary of Films Generated Under Different Experimental Conditions via AACVD and Their Resulting WCAs and Transmittance

Film	Deposition temperature/°C	Deposition time/min	WCA/°	Transmittance/%
ES/0.6PDMS^a^	360	40	155 ± 2	5
ES/0.6PDMS/0.2SiO_2_^b^	360	40	159 ± 2	9
ES/0.6PDMS/0.3SiO_2_	360	40	162 ± 0	11
ES/0.6PDMS/0.4SiO_2_	360	40	162 ± 1	15
ES/0.6PDMS/0.6SiO_2_	360	40	165 ± 3	19
ES/0.6PDMS/0.8SiO_2_	360	40	166 ± 1	27
ES/0.6SiO_2_^c^	360	40	119 ± 0	71
ES/0.2PDMS/0.6SiO_2_	360	40	158 ± 7	35
ES/0.4PDMS/0.6SiO_2_	360	40	160 ± 1	25
ES/0.8PDMS/0.6SiO_2_	360	40	167 ± 3	13
ES/0.6PDMS/0.6SiO_2_	360	15	114 ± 3	64
ES/0.6PDMS/0.6SiO_2_	360	20	117 ± 2	62
ES/0.6PDMS/0.6SiO_2_	360	25	138 ± 1	44
ES/0.6PDMS/0.6SiO_2_	360	30	145 ± 1	44
ES/0.6PDMS/0.6SiO2	360	35	164 ± 2	21
0.6PDMS/0.6SiO_2_^d^	360	40	163 ± 2	29

a Precursor containing 0.6 g of PDMS deposited on the ES-pretreated glass.

b Precursor containing 0.6 g of PDMS and 0.2 g of SiO_2_ deposited on the ES-pretreated glass.

c Precursor containing 0.6 g of SiO_2_ deposited on the ES-pretreated glass.

d Precursor containing 0.6 g of PDMS and 0.6 g of SiO_2_ deposited on the untreated glass.

## Characterization

The surface morphology of the samples was determined using scanning electron microscopy (SEM) with an accelerating voltage of 10–15 kV. The films were sputtered with gold to improve the electrical conductivity before imaging. The particle size was estimated using ImageJ version 1.54s. The chemical composition of the films was studied using attenuated total reflection-Fourier transform infrared spectroscopy (ATR-FTIR) in the range of 400–4000 cm^–1^, and X-ray photoelectron spectroscopy (XPS) was carried out on a Thermo Scientific K-alpha photoelectron spectrometer with monochromatic Al–Kα radiation. The peaks in the XPS spectra were analyzed using Avantage V5 software. Ultraviolet–visible (UV–vis) spectra were recorded in the range of 400–800 nm using a Shimadzu UV-2700 spectrophotometer. The surface roughness Sq (root-mean-square height) was estimated using the Keyence optical microscope at 1500× magnification. The measurements were taken at three different positions of each film, and the mean with the standard deviation was calculated. The surface energy was determined by measuring the contact angles with water and diiodomethane and then calculated using the Owens, Wendt, Rabel, and Kaelble (OWRK) method.

The water contact angle (WCA) of the coatings was determined by using a Kruss DSA25E drop shape analyzer. Approximately 5 μL deionized (DI) water droplets were dispensed at 6 different positions of each film. The WCA was determined using the Young–Laplace equation. The sliding angle (SA) was determined using the tilted drop approach with ∼15 μL of DI water droplets dropped from a height of 4 cm onto the middle of the film. The difference between the advancing and receding angles was used to determine the contact angle hysteresis (CAH).

## Robustness and Self-Cleaning Testing

### Tape Peel Test

Scotch Magic Tape was repeatedly attached to and removed from the film 400 times. WCAs and SAs of the films were measured throughout the test to determine the retention of the SH property.

### Organic Solvent Test

Pieces of the SH films were immersed in ethanol, diethyl ether, and toluene for 5 h with WCAs measured every 1 h and SAs measured at the end of immersion.

### Thermal Stability Test

The films were heated in a furnace at 300 °C for 5 h and at 400 °C for an additional 5 h, with cooling to room temperature after each heating cycle. The WCAs and SAs were then measured.

### UV Stability Test

The SH films were exposed to ultraviolet light (UV) at 365 nm, 258 mW cm^–2^ at room temperature for 2 weeks with WCAs measured every odd day and SAs assessed on days 5, 9, and 14.

### Self-Cleaning Abilities

To determine the self-cleaning potential of the films, gold glitter was used to represent “the contaminant” and to coat the surface. Subsequently, several water droplets were dropped onto the film. To visualize the water repellency of the films, a methylene blue solution was dispensed onto the coating at a tilt angle of 20°. In both tests, photographs were taken before, during, and after the test.

## Results and Discussion

**Figure 2:**
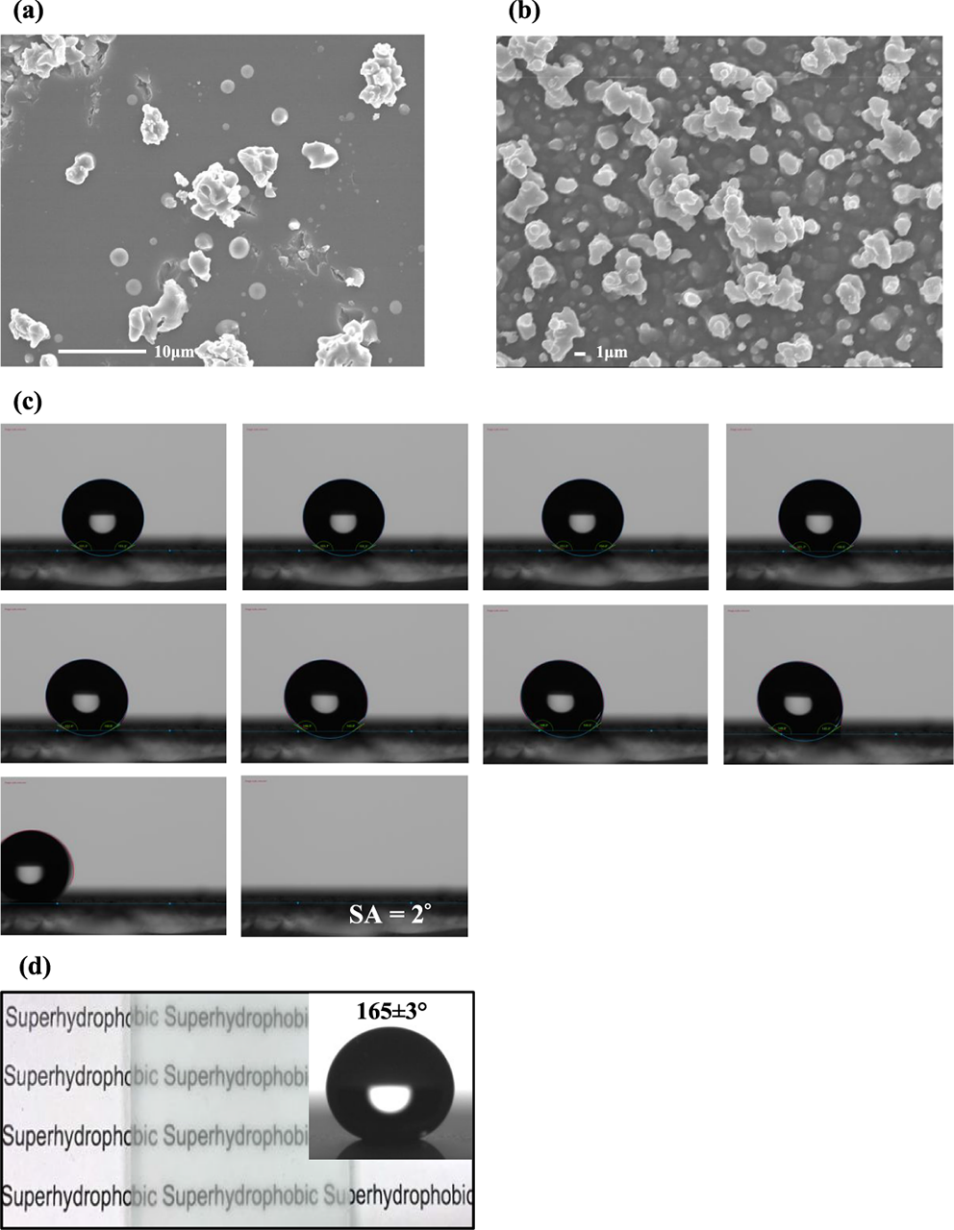
SEM images of (a) ES on glass and (b) an ES/0.6PDMS/0.6SiO_2_ film (deposited at 360 °C for 40 min). Optical images of (c) SAs and (d) visible transparency and WCA for the ES/0.6PDMS/0.6SiO_2_ film.

To produce robust and fluorine-free SH films, ES, PDMS, and SiO_2_ NPs were used in this study, as illustrated in Figure 1. As shown in Table 2, the plain glass substrate had a WCA of 68°. After pretreating the glass with the ES, the WCA of the coating increased to 88 ± 2°, indicating its hydrophilic nature, with the SEM image shown in Figure 2a. It was expected that a layer of ES deposited onto a glass substrate would be uniform and smooth. Ghanbari et al. deposited GPTMS onto a glass substrate and obtained a smooth layer of the silane.^37^ Previously, Sarkari et al. deposited GLYMO by various deposition techniques and demonstrated a relatively conformal coating.^38^ Therefore, the brushing method and later exposure to a temperature of 360 °C deposited within this paper may have led to strong oligomerization of the silane, engendering roughness as demonstrated by the top-down and side-on SEM images (Figure 2a and S5) and relatively high water contact angles (Table 2). Depositing PDMS onto the ES-pretreated glass produced a superhydrophobic surface with a WCA of 155 ± 1°and a SA of 5°. The SEM image in Figure S1 confirmed that a rough surface morphology was obtained due to the polymer aggregation that occurred during the AACVD process. This rough surface generated by the PDMS via AACVD, in combination with its inherent hydrophobicity, provided the film with water-resistant properties.^33,39^ Although the ES/PDMS coating had some surface roughness (Sq = 0.45 ± 0.04 μm), the roughness obtained was not high enough to achieve excellent water repellency. It was found that adding SiO_2_ NPs into the PDMS precursor solution greatly increased the roughness to 1.01 ± 0.07 μm, resulting in excellent superhydrophobicity (WCA = 165 ± 3°, SA = 2°, Table 2 and Figure 2c,d), which was comparable to the water contact angles of fluorinated SH films reported in the literature.^9,11,30,40^

**Table 2:** Summary of WCAs, SAs, CAH, and Roughness Values (Sq) for Plain Glass, ES, ES/PDMS, and ES/PDMS/SiO_2_ Films

Film	Deposition temperature/°C	Deposition time/min	WCA/°	SA/°	CAH/°	Sq/μm
Plain glass	N/A	N/A	68 ± 0	N/A	N/A	N/A
ES on glass	N/A	N/A	88 ± 2	N/A	N/A	N/A
ES/0.6PDMS	360	40	155 ± 1	5 ± 1	24 ± 5	0.45 ± 0.04
ES/0.6PDMS/0.6SiO2^a^	360	40	165 ± 3	2 ± 1	14 ± 2	1.01 ± 0.07

a This film is the same as the ES/0.6PDMS/0.6SiO_2_/360 °C/40 min film.

The lower CAH for the ES/PDMS/SiO_2_ film further proved that the presence of SiO_2_ roughened the surface and thus enhanced the superhydrophobicity.^41^ We can observe that the coating consisted of differently sized and nonuniform microscale clusters that were surrounded by numerous nanosized silica particles (Figure 2b). These microscale clusters were formed due to the aggregation of SiO_2_ NPs that were tightly adhered to the PDMS agglomerates during the deposition process. Such textured surface containing micro/nanostructures and a low surface energy reagent (PDMS) possessed superior water repellency.

Since the deposition of PDMS and SiO_2_ NPs on the ES-pretreated glass resulted in a well-adhered film with excellent superhydrophobicity and higher transparency compared to the ES/PDMS film, this combination of precursors was chosen to study the effect of deposition temperatures on the resulting films. The results indicated that the deposition temperature plays an important role in the surface morphology. As shown in Figure S2, the number of particles of different sizes and particle density increased with increasing temperatures (300–360 °C). However, further increasing the temperature to 380 and 400 °C caused a decrease in the number and size of particles. The results of wettability and Sq values of these films showed that WCA, SA, and surface roughness were optimized at 360 °C (Figure S2). Therefore, all subsequent depositions were carried out at this temperature. The following studies involved investigating the effect of varying the *x*SiO_2_ (where *x* = 0, 0.2, 0.3, 0.4, 0.6, and 0.8 g) and *x*PDMS (where *x* = 0, 0.2, 0.4, 0.6, and 0.8 g) loadings in the precursor mixture as well as the deposition time (15, 20, 25, 30, 35, and 40 min). All samples were characterized using a range of techniques including SEM, optical microscopy, UV–vis spectroscopy and contact angle goniometry to determine the optimum parameters for producing translucent and superhydrophobic ES/PDMS/SiO_2_ coatings.

**Figure 3:**
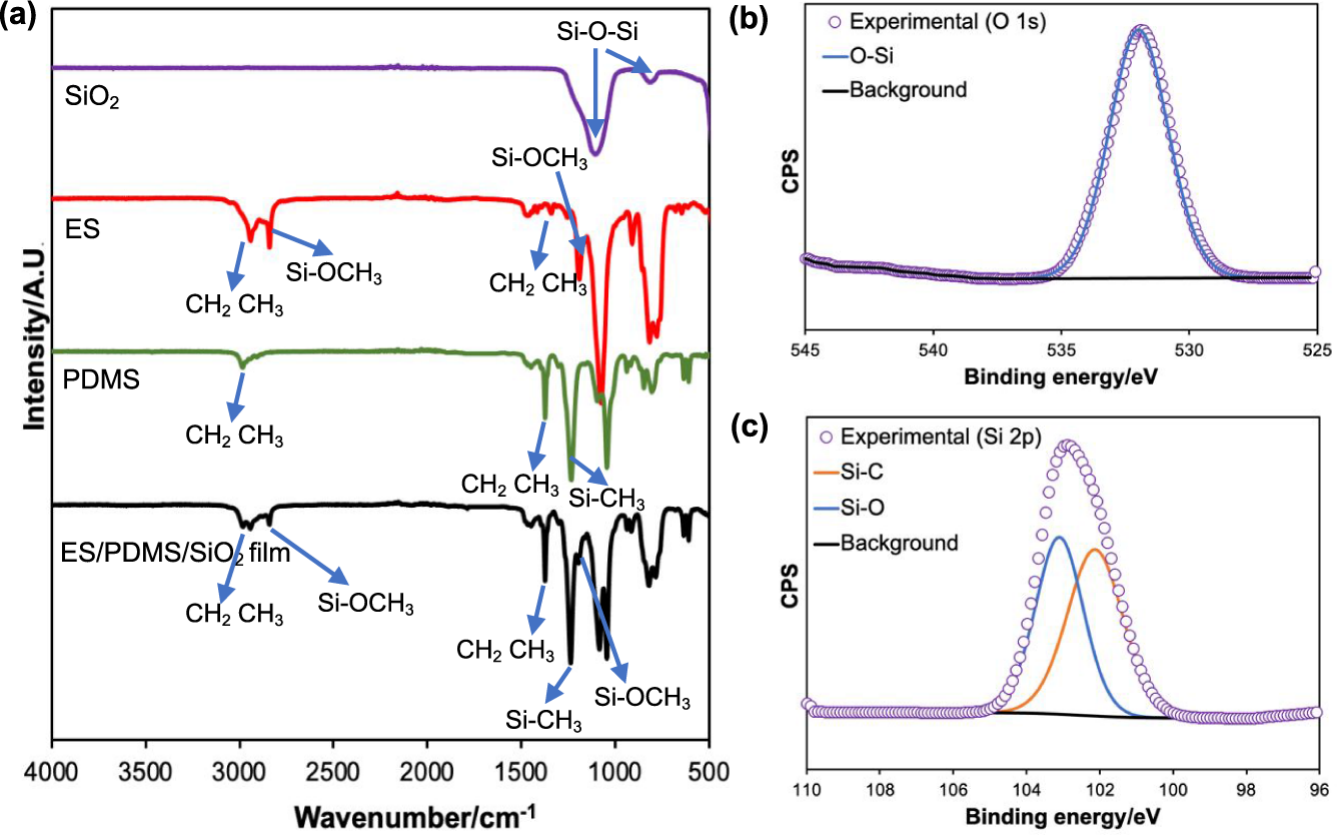
(a) FT-IR spectra of SiO_2_, ES, and PDMS starting materials and the ES/PDMS/SiO_2_ film. XPS data showing (b) O 1s and (c) Si 2p spectra for the ES/PDMS/SiO_2_ surface.

The chemical composition of the ES/PDMS/SiO_2_ film was identified by FT-IR. In Figure 3a, the peaks at 3007–2914 cm^–1^ were assigned to the stretching vibrations of CH_2_ and CH_3_ groups of PDMS and ES, and the small peaks around 1450 and 1350 cm^–1^ were associated with their bending vibrations.^21^ The sharp peak at 1250 cm^–1^ was due to the sp^3^ C–H deformation in Si-CH_3_ of PDMS.^30^ The signals observed at 2841 and 1193 cm^–1^ were due to the Si-OCH_3_ group of ES.^42^ The strong peaks at around 1083 and 800 cm^–1^ represented the Si–O–Si asymmetric and symmetric stretching vibrations, respectively, of ES, PDMS, and SiO_2_.^30^ Since the chemicals used have the same components (including C, H, O, and Si), each individual peak could be associated with more than one reagent. All peaks shown in the IR spectrum were consistent with those in the literature.

XPS was employed to understand the surface chemistry of the ES/PDMS/SiO_2_ film. The O 1s spectrum contained a signal at 531.9 eV (Figure 3b), corresponding to the Si–O bond of PDMS and SiO_2_. Since XPS can only detect the sample surface, the peak associated with the O–C bond of ES (base layer) was not observed. Figure 3c (Si 2p) showed double peaks at 102.0 and 103.0 eV, indicating the Si–C bond of PDMS and the Si–O bond of PDMS and silica NPs, respectively. All binding energies reported were in agreement with ref. (30). As expected, the XPS survey spectrum contained only C, O, and Si signals, indicating no contaminants in the film (Figure S3).

**Figure 4:**
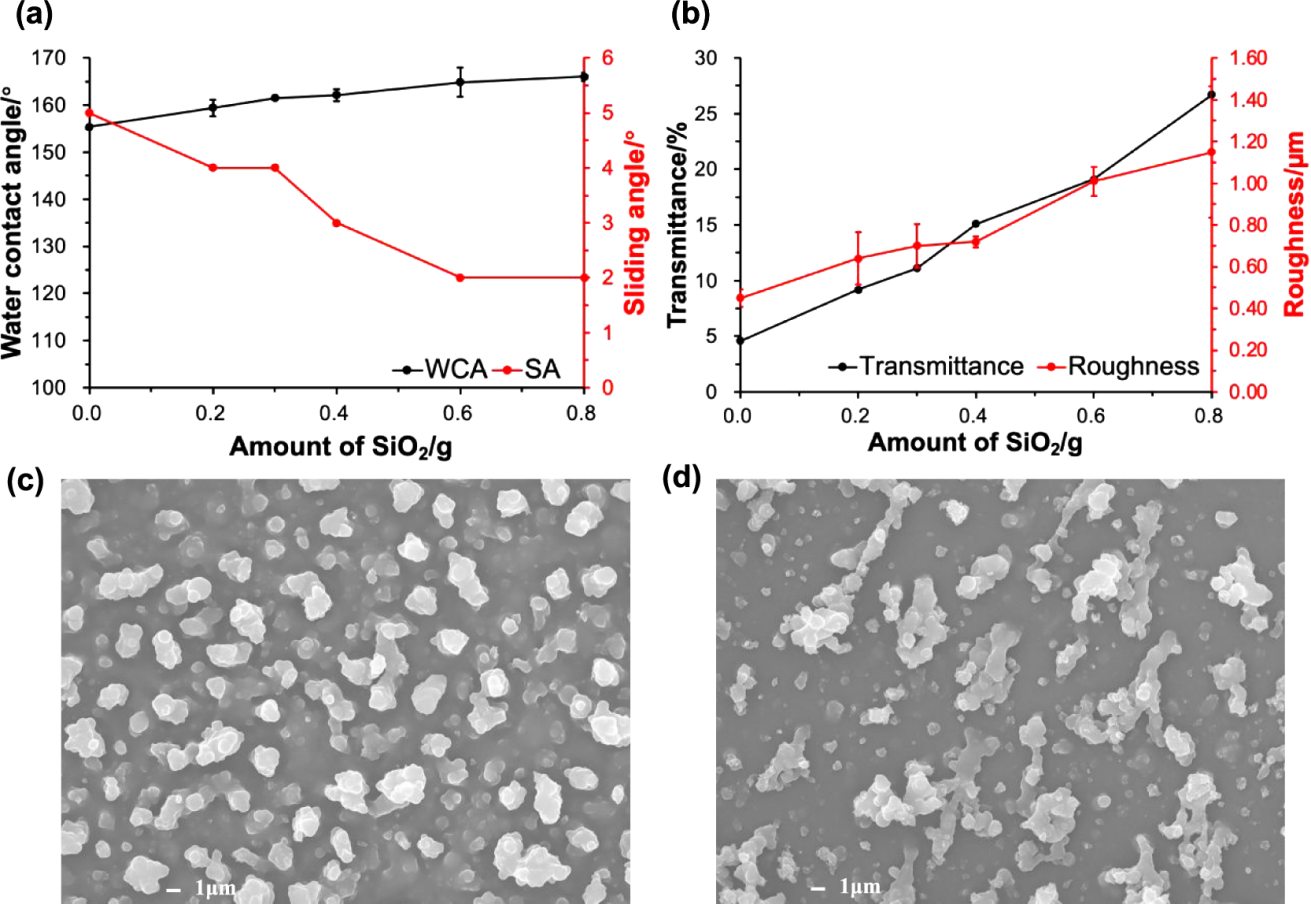
(a) WCAs and SAs and (b) transmittance and surface roughness of ES/0.6PDMS/xSiO_2_ films (*x* = 0, 0.2, 0.3, 0.4, 0.6, and 0.8). SEM images of (c) ES/0.6PDMS/0.2SiO_2_ and (d) ES/0.6PDMS/0.8SiO_2_ films, showing an increase in surface roughness and porosity with increasing SiO_2_ content.

To investigate the effects of changing the SiO_2_ NPs loading on the resulting films, 0, 0.2, 0.3, 0.4, 0.6, and 0.8 g of SiO_2_ were added to the solution containing 0.6 g of PDMS, named ES/0.6PDMS/xSiO_2_ films. All depositions were performed at 360 °C. It was found that superhydrophobicity increased gradually with the increase of the SiO_2_ content, with the highest WCA (166 ± 1°) and the lowest SA (2°) observed with 0.8 g of SiO_2_ (Figure 4a). This was confirmed by the roughness value given in Figure 4b, with the ES/0.6PDMS/0.8SiO_2_ film exhibiting the largest Sq value (1.15 ± 0.31 μm), a measure of the surface height variations.^43^ The increase in superhydrophobicity and roughness can be explained by the SEM images. As can be seen in Figure S1, the ES/0.6PDMS film consisted of some spherical particles that aggregated into bigger and nonuniform clusters, resulting in an increase in the surface roughness. However, the clusters formed were similar in size ranging from 500 nm to 3.2 μm, leading to small surface height variations (Sq = 0.45 ± 0.04 μm), and therefore giving a relatively flat surface and low superhydrophobicity (WCA = 155 ± 2°). The surface morphology changed when SiO_2_ was added to the PDMS solution. As shown in Figure 4c,4d, both films verified that the use of a combination of PDMS and silica NPs resulted in a rough surface containing micro- and nanosized particles of different sizes, which corresponds with the morphology required for superhydrophobicity as reported in refs. (12, 30, 44).When the SiO_2_ loading was increased to 0.8 g, a large number of silica NPs aggregated and adhered to the PDMS, which significantly increased the cluster size. These features led to the largest number and range of particle sizes for the ES/0.6PDMS/0.8SiO_2_ film, with diameters between 300 nm and 10.2 μm, thus achieving a highly rough surface with the greatest height variations and the best superhydrophobicity.

In addition, SiO_2_ also played a role in improving the transparency (Figure 4b). The SEM images in Figure 4c,4d showed that increasing the SiO_2_ loading resulted in an increase in the particle size but a decrease in the particle density per unit area. The large spatial separation of micro- and nanosized particles on the ES/0.6PDMS/0.8SiO_2_ surface created some uncovered spaces and pores, thus facilitating visible light transmission. In contrast, as shown in Figures S1 and 4c, there was almost no uncovered space on the films with no or low silica content, resulting in poor transparency. This agrees well with the findings by Zhuang et al., who showed that the film with low-density micro/nanostructures greatly enhanced the light penetration compared to the film with high-density structures.^44^ Although the highest water repellency and optical transmittance were achieved using 0.8 g of SiO_2_, its powdered nature made the film less robust and adherent, as the film could be wiped off with a finger. To obtain well-adhered, translucent, and superhydrophobic films, 0.6 g of SiO_2_ was taken as a compromise between these three properties.

**Figure 5:**
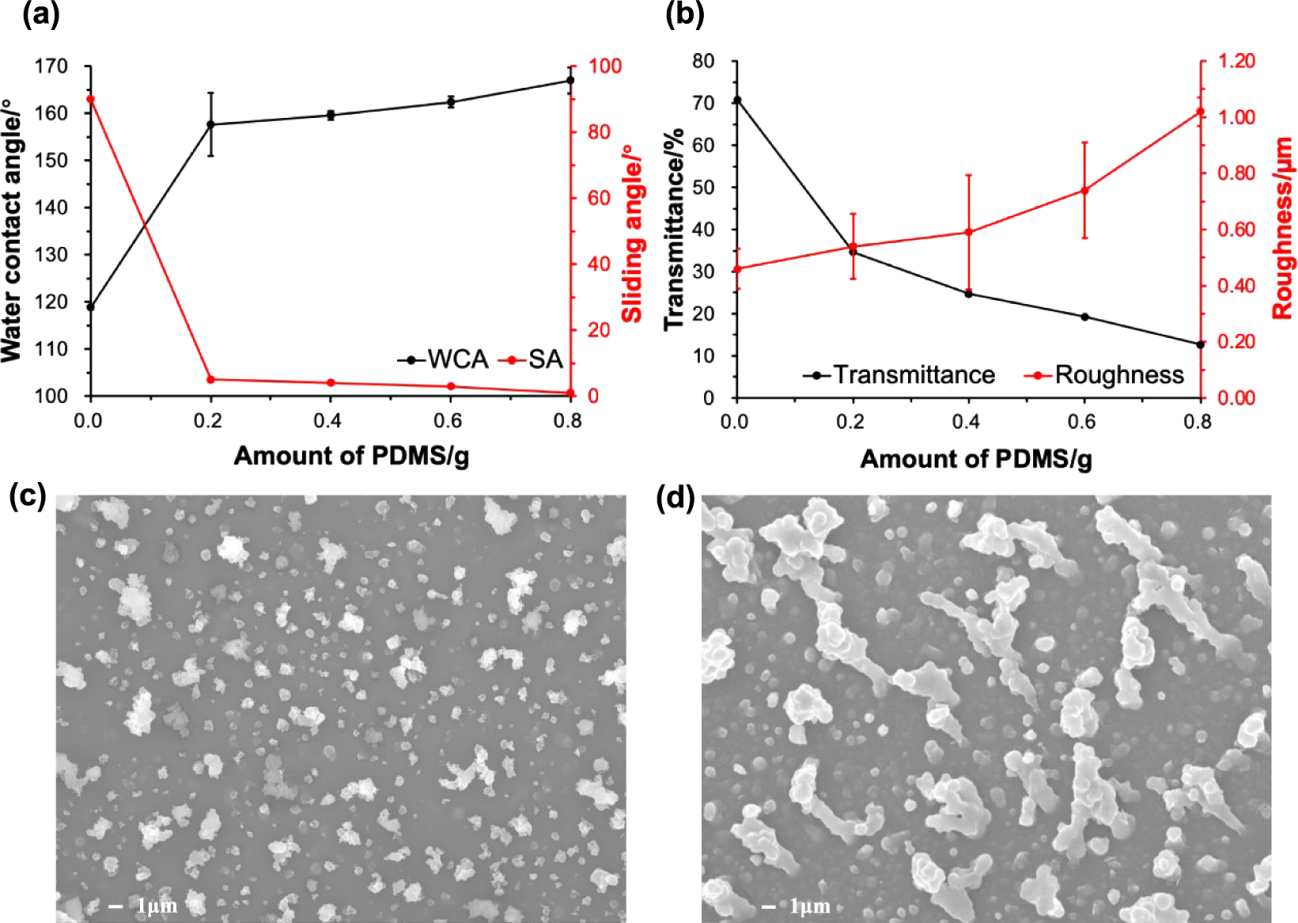
(a) WCAs and SAs and (b) transmittance and surface roughness of ES/xPDMS/0.6SiO_2_ films (*x* = 0, 0.2, 0.4, 0.6, 0.8). SEM images of (c) ES/0.2PDMS/0.6SiO_2_ and (d) ES/0.8PDMS/0.6SiO_2_ films showing an increase in surface roughness with increasing PDMS content.

The PDMS loading was also varied (0, 0.2, 0.4, 0.6, and 0.8 g) to explore its influence on superhydrophobicity and transparency, named ES/xPDMS/0.6SiO_2_ films. As before, the deposition temperature was kept constant at 360 °C to minimize the variability of the resulting films. Here, PDMS played a role in reducing the surface energy and increasing the roughness. Figure 5a shows that superhydrophobicity increased with an increasing amount of PDMS. Due to the lack of a low surface energy reagent, the ES/0.6SiO_2_ film showed poor water repellency (WCA = 119°). The water droplets were unable to roll off the surface even at a tilt angle of 90°, which was an example of a sticky hydrophobic surface.^45^ After the addition of 0.2 g of PDMS, the film became superhydrophobic, and the WCA increased significantly to 158 ± 7°. The water droplets easily slid off at a tilt angle of 5°, indicating a small attraction between the liquid and the surface, thus describing the Cassie–Baxter state.^45^ These results suggested that the presence of the low surface energy reagent (PDMS) was important to achieve Cassie–Baxter behavior. Further addition of PDMS increased superhydrophobicity slightly, with the WCA and SA reaching 167 ± 3° and 1°, respectively, at the highest PDMS loading. The effect of PDMS on the water repellency was in line with work by Kalmoni et al., who showed that an initial increase in the concentration of PDMS greatly improved the superhydrophobicity, while further addition of PDMS increased WCAs slightly.^30^ This result indicated that PDMS also contributed to the surface roughness, which was confirmed by the roughness values which increased Figure 5b from 0.46 ± 0.07 μm with no PDMS content to 1.02 ± 0.05 μm for 0.8 g of PDMS. The SEM images in Figure 5c,5d indicated that PDMS enhanced the microscale roughness. This was because PDMS has high viscosity, allowing particles to aggregate and stick together during the AACVD process.^33^ The ES/0.8PDMS/0.6SiO_2_ coating consisted of some large agglomerates with diameters between 6.8 and 12.2 μm, which considerably increased the particle size range (from 440 nm to 12.2 μm) and hence the roughness. Conversely, due to the low PDMS loading, the clusters formed on the ES/0.2PDMS/0.6SiO_2_ surface were relatively small, resulting in similar particle sizes (from 270 nm to 3.5 μm). Therefore, micro/nanostructures with a large size range and excellent SH properties can be achieved in combination with SiO_2_ NPs and high PDMS content.

As can be seen from Figure 5b, the ES/0.8PDMS/0.6SiO_2_ coating had the lowest transmittance (13%) due to the high surface roughness and large microscale clusters, causing serious light scattering. The above observation was confirmed by the Mie theory that describes the increase in light scattering with increasing particle size.^46^ Therefore, all subsequent depositions were performed with 0.6 g of PDMS to balance the transparency and superhydrophobicity.

**Figure 6:**
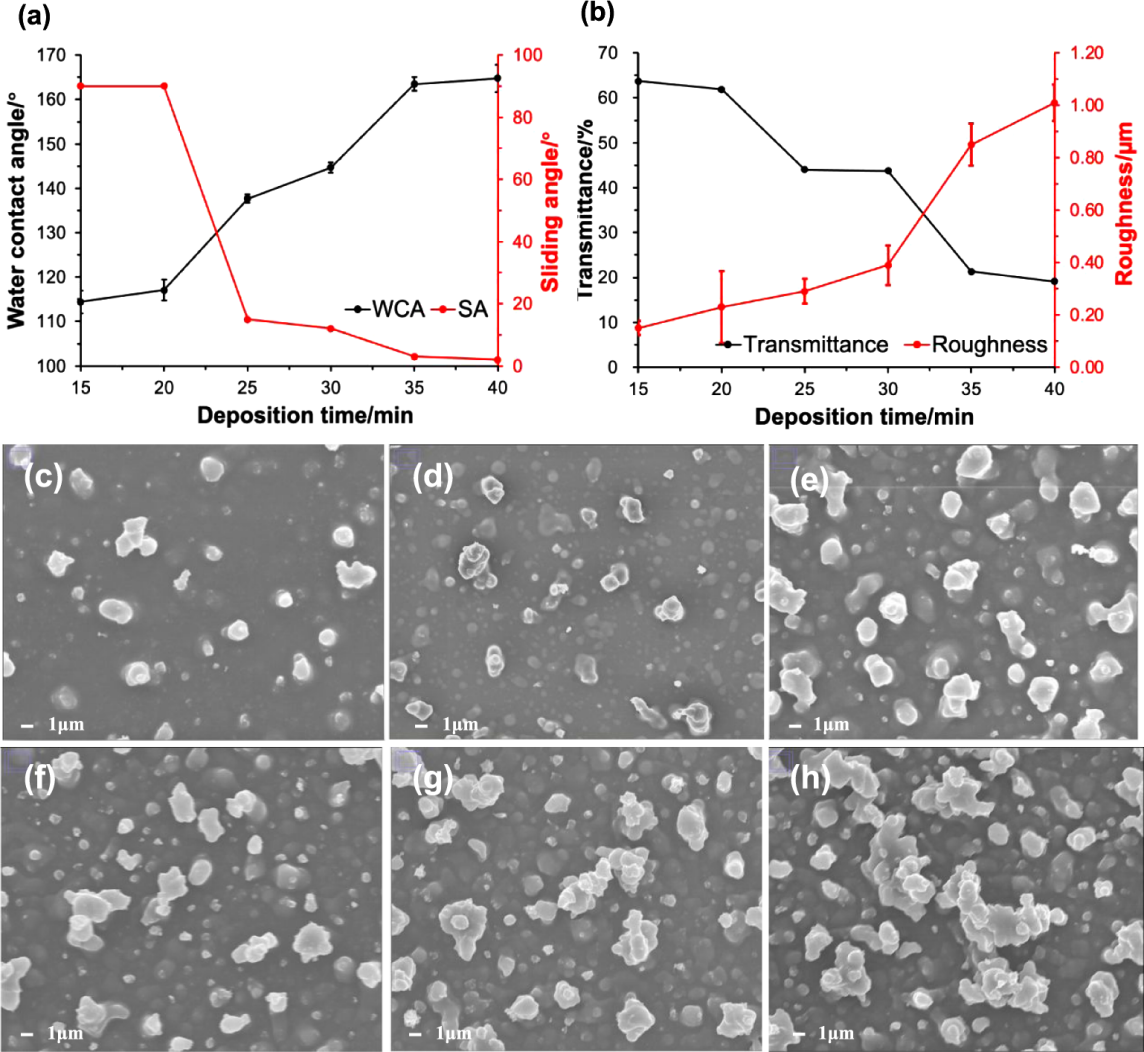
(a) WCAs and SAs and (b) transmittance and surface roughness of ES/0.6PDMS/0.6SiO_2_ films deposited at 15, 20, 25, 30, 35, and 40 min. Top-down SEM images of ES/0.6PDMS/0.6SiO_2_ films deposited at (c) 15, (d) 20, (e) 25, (f) 30, (g) 35, and (h) 40 min.

The effect of varying the deposition time (15, 20, 25, 30, 35, and 40 min) on the superhydrophobicity, thickness, and transparency of the ES/0.6PDMS/0.6SiO_2_ film was investigated at a constant deposition temperature (360 °C). As shown in Figure 6a, increasing the deposition time led to an increase in the water repellency of the films, with the highest WCA (165 ± 3°) and the lowest SA (2°) being achieved at 40 min. The top-down SEM images in Figure 6c**–**h confirmed this trend, which illustrated that the ES/0.6PDMS/0.6SiO_2_/40 min film exhibited the largest range of particle size (between 300 nm and 8.3 μm), the greatest number of particles of different sizes, and the highest particle density among all the films, thus giving the highest Sq value (1.01 ± 0.07 μm) (Figure 6b). In this study, the transition from the Wenzel state to the Cassie–Baxter state was evident and was easily achieved by extending the deposition time. When the deposition time was less than 20 min, the water droplets did not roll off the surface, even at a tilt angle of 90°. Although a low surface energy was achieved (7.1 mJ/m^2^) within 20 min, the insufficient formation of micro/nanostructures in a very short period of time and the relatively thin layer of coating obtained led to poor water repellency. Increasing the deposition time to 35 min resulted in a SH surface (WCA = 164 ± 2°). The highly rough surface with an even lower surface energy (1.7 mJ/m^2^), trapped air bubbles beneath the water droplets and reduced the attraction between the liquid and the substrate, minimizing the contact area and adhesion between these two phases, hence achieving Cassie–Baxter behavior. Further extending the reaction time from 35 to 40 min gave similar WCAs as most of the precursors had been aerosolized and deposited, resulting in no significant change in surface morphology.

Figure 6b shows the relationship between transparency and deposition time. The ES/0.6PDMS/0.6SiO_2_/15 min film showed the highest transmittance (64%) but the lowest WCA (114 ± 3°). The opposite was found for the ES/0.6PDMS/0.6SiO_2_/40 min coating. The side-on SEM images in Figure S4 indicated that the film became thicker when the reaction time was extended from 15 to 40 min, which caused a decrease in transparency. Meanwhile, the rough surfaces consist of dense and large particles that can increase light scattering, which can be verified by top-down SEM images. Consistent with the literature, Li et al. reported that the longer the deposition time, the thicker the films, the denser the particles, the rougher the surfaces, and so the lower the transparency.^12^

Various approaches of ES pretreatment were employed to investigate their effects on the obtained films. However, it was found that multiple ES layers could decrease the WCA of the films. In this work, by applying 2 layers of the ES onto the glass substrate, the resulting film became hydrophobic (WCA = 139°). In addition, attempts were made to deposit the ES base coating via AACVD rather than brush-coating. However, at a low temperature (200 °C), the precursor leaked from the baffle as the low temperature was not high enough to keep the solvent in the gas phase and/or to transport the aerosol into the heated chamber. Furthermore, incorporating the ES precursor into the PDMS/SiO_2_ precursor mixture caused the subsequent film to become hydrophobic rather than SH due to its inherent hydrophilic nature, with lower hydrophobicity observed at higher contents, giving WCAs of 106°, 102°, and 98° for 0.3, 0.6, and 1.8 mL of ES, respectively. The ES has a high surface tension (42.5 mN/m) since hydrophilic surfaces generally have surface tensions of around 45 mN/m, leading to a strong attraction between the surface and the liquid.^47^ In addition, as seen in Figure 2a, the ES coating is smooth with fewer particles compared to the SH film (from 2.1 to 7.0 μm). This smooth microstructure and high surface tension potentially contributed to the hydrophilic/borderline hydrophobic nature of the ES basecoat (88°) and a thicker coating had a greater effect on the chemistry of the SH material. A smaller decrease in the WCA was obtained when ES coatings were used as the base layer rather than when the ES was incorporated into the precursor mixture, which could be because the former had a smaller effect on the surface.

In this work, the depositions of ES/PDMS/SiO_2_ films were carried out under a range of reaction conditions to improve optical transmittance. It is well-known that superhydrophobicity and transparency are competing factors.^46^ An increase in the WCA means that a rougher surface morphology is obtained, which makes it difficult for light to penetrate and thus reduces the transparency of the film. This was in line with the competing relationship between transmittance and roughness observed for the films deposited at various temperatures, PDMS loadings, and deposition times (Figures S2, 5b, and 6b). However, an opposite trend was obtained for the films fabricated with different SiO_2_ loadings, with both the transmittance and roughness increasing (Figure 4b), suggesting the potential ability of silica NPs to improve the water repellency and transparency. SEM images in Figure 4c,4d confirmed this finding, which showed that increasing the SiO_2_ content led to an increase in the particle size and surface roughness, but there was more uncovered space and porosity in the film, thus allowing more light to pass through. Nevertheless, the coatings displayed lower transmittance compared to the fluorinated SH films due to the presence of large microparticles.^40,44^ The films grown in our study consisted of particles of approximately 300 nm–8 μm in size, whereas the particle size of fluorinated films was concentrated in the 300 nm–2 μm range, thereby making the improvement of SiO_2_ less effective.^44^

## Robustness Testing

The robustness of SH films is one of the most important features in real-world applications. The optimum film obtained in this work (ES/0.6PDMS/0.6SiO_2_/360 °C/40 min) was selected to investigate the durability. It is important to add that film ES/0.6PDMS/0.6SiO_2_/360 °C/40 min is the same as film ES/0.6PDMS/0.6SiO_2_, and hence, the latter will be used in forthcoming discussions.

For comparison, the 0.6PDMS/0.6SiO_2_/360^◦^C/40 min film (shortened to film 0.6PDMS/0.6SiO_2_) was also studied, with the SEM image shown in Figures 6h and S6. Table 3 compares the superhydrophobicity of these two films, including WCAs, SAs, and CAH. Both surfaces exhibited similar wettability, with WCAs > 160°, SAs < 4°, and CAH around 15°. The side-on SEM images (in Figure S5) showed the thickness of the ES, ES/0.6PDMS/0.6SiO_2_, and 0.6PDMS/0.6SiO_2_ films to be 19.8, 24.4, and 19.2 μm, respectively. Since the film without the ES underlayer was thinner, the transmittance of 0.6PDMS/0.6SiO_2_ (29%) was slightly higher than that of ES/0.6PDMS/0.6SiO_2_ (19%). However, the film thickness was beneficial for robustness, and the following robustness tests proved that pretreating the glass with ES greatly improved the durability of the films.

**Table 3:** Summary of WCAs, SAs, CAHs, and Transmittance for ES/0.6PDMS/0.6SiO_2_ and 0.6PDMS/0.6SiO_2_ Films

Film	Deposition condition		Superhydrophobicity			Transmittance/%
	Temperature/°C	Time/min	WCA/°	SA/°	CAH/°	
ES/0.6PDMS/0.6SiO2	360	40	165 ± 3	2 ± 1	14 ± 2	19
0.6PDMS/0.6SiO_2_	360	40	163 ± 2	3 ± 1	15 ± 1	29

**Figure 7:**
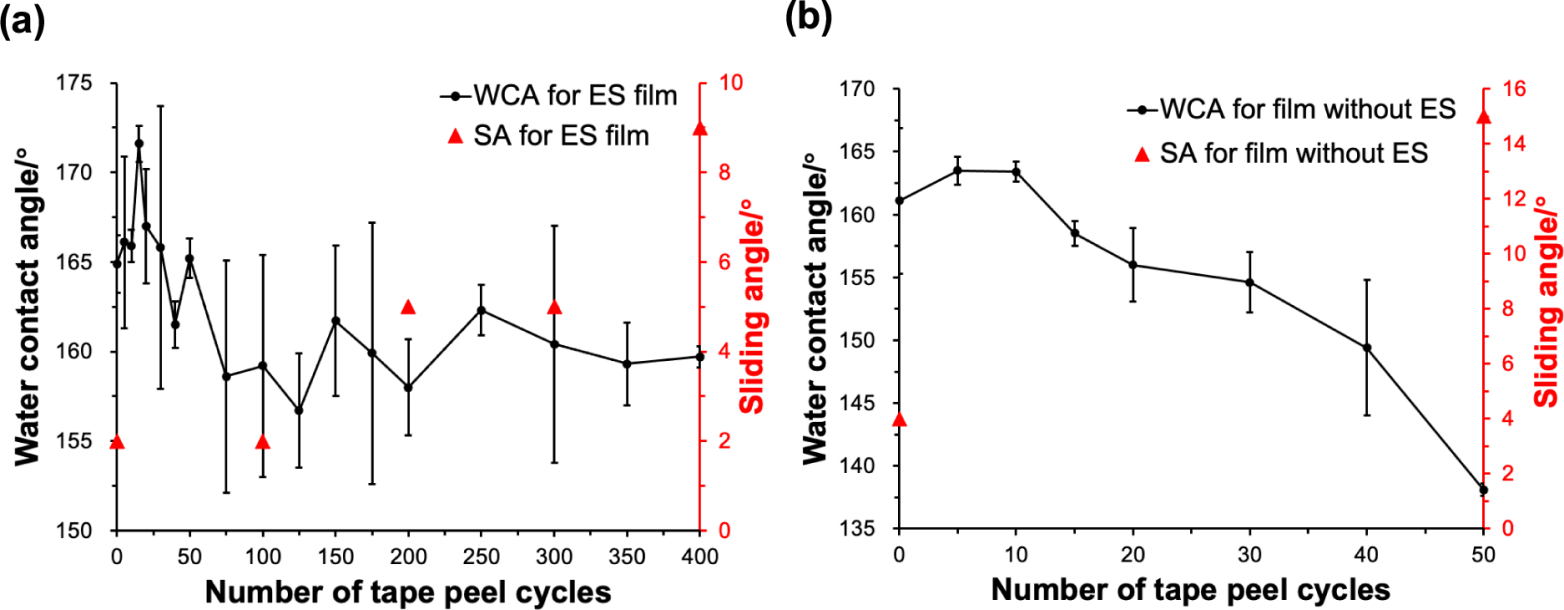
WCAs and SAs for (a) ES/0.6PDMS/0.6SiO_2_ and (b) 0.6PDMS/0.6SiO_2_ films after different tape peel cycles.

Various methods were used to evaluate the robustness of the films, including tape peel cycles, immersion of the films in solvents of different polarities, heating to high temperatures, and exposure to UV light. The adhesion between the film and the substrate was determined by the tape peel test (Figure 7). It was found that the ES/0.6PDMS/0.6SiO_2_ film still retained its superhydrophobicity even after 400 tape peel cycles with a WCA of 160 ± 2° and a SA of 9°, suggesting excellent adhesion of coating to the substrate. For comparison, experiments were also carried out on the 0.6PDMS/0.6SiO_2_ film. The WCAs decreased significantly from 160 ± 6° to 138 ± 1° after 50 cycles, showing hydrophobicity and poor adhesion.

**Figure 8:**
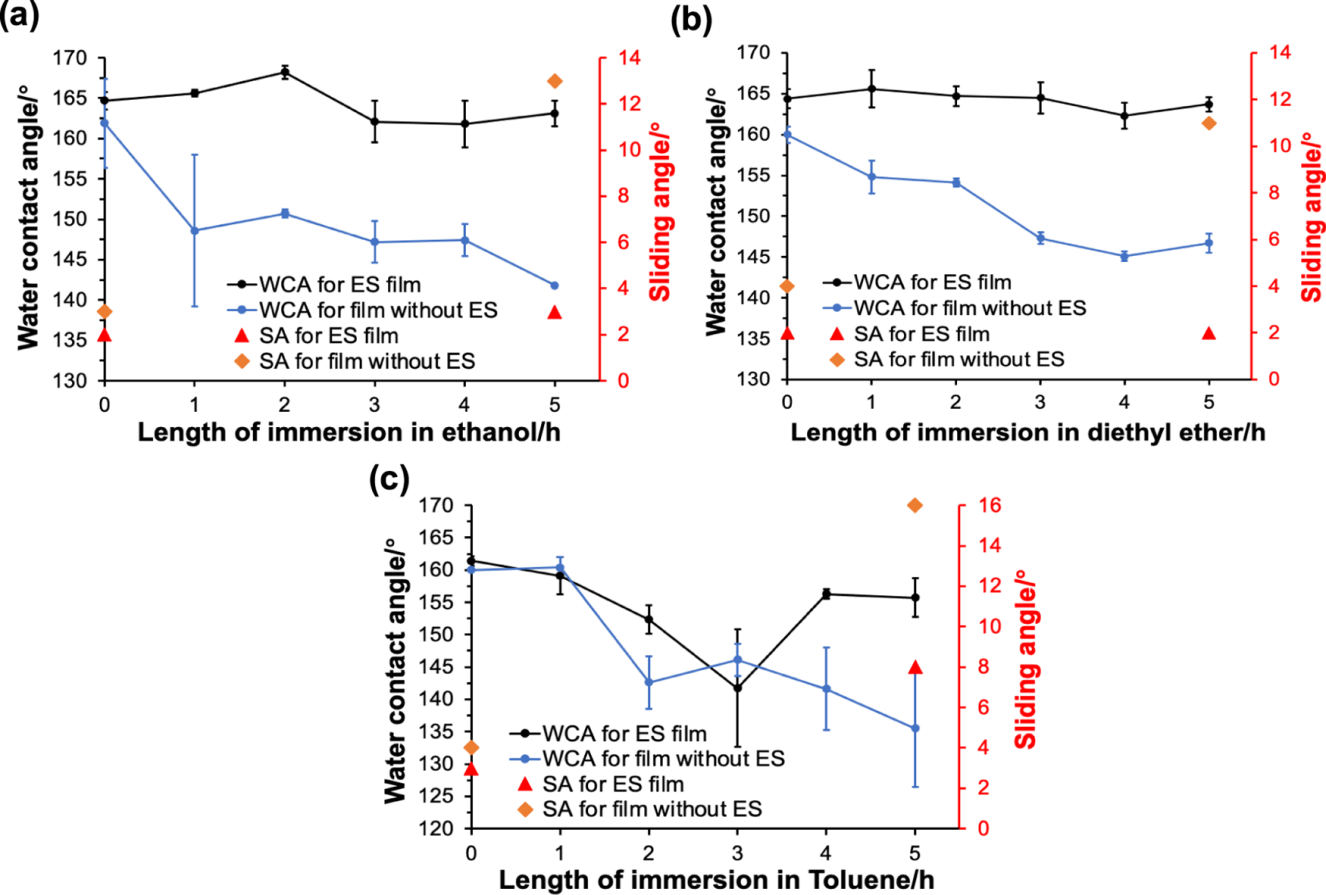
WCAs and SAs for ES/0.6PDMS/0.6SiO_2_ and 0.6PDMS/0.6SiO_2_ films during 5 h of immersion in (a) ethanol, (b) diethyl ether, and (c) toluene.

The stability of films exposed to organic solvents of different polarities was estimated (ethanol, diethyl ether, and toluene). As shown in Figure 8, the WCAs and SAs of ES films immersed in ethanol and diethyl ether were nearly unchanged after 5 h, with WCAs > 160° and SAs < 3°, suggesting consistent superhydrophobicity. The ES film exposed to toluene showed a decrease in the WCA during the test, but its water repellency was recovered with WCAs returning to >150°, which could be caused by toluene not being completely removed from the glass during the measurement. For comparison, the same tests were performed on the 0.6PDMS/0.6SiO_2_ films. None of the films retained their superhydrophobicity after 5 h of immersion, with WCAs falling to 142 ± 0°, 147 ± 1°, and 136 ± 9° for ethanol, diethyl ether, and toluene, respectively. The poor adhesion of the PDMS/SiO_2_ coatings led to their surface structures being washed out by the solvents.

The excellent adhesion of the ES/PDMS/SiO_2_ film can be attributed to the following reasons. Firstly, the ES contains a reactive epoxide group, forming strong hydrogen bonds not only with the hydroxyl groups on the glass surface but also with the Si–O groups of PDMS and SiO_2_ NPs via a ring-opening reaction, which greatly improved the bonding strength between the coating and the glass substrate. The additional plasma treatment increased hydroxyl groups on the glass surface, leading to better adhesion of the ES to the substrate.^24^ Secondly, there are three methoxy groups in the ES. The oxygen atoms in the methoxy groups can form hydrogen bonds with the surface OH groups and the C–H bond of PDMS, therefore further enhancing the interaction between the film and the surface.^48^ Thirdly, the elastic PDMS polymer also contributed to the overall robustness as it can be cured to form a cross-linked rubber with excellent durability.^49,50^

Table 4 highlights the stability of the ES/0.6PDMS/0.6SiO_2_ film at high temperatures. After heating at 300 and 400 °C for 5 h, the superhydrophobicity of the ES/0.6PDMS/0.6SiO_2_ coating was unaffected, with WCAs of 164 ± 2° and 166 ± 2°, respectively. In contrast, the WCA of the 0.6PDMS/0.6SiO_2_ film decreased significantly to 118 ± 4° after heating at 400 °C, which was similar to that of the ES/0.6SiO_2_ film (119°, Table 1). This was due to the thermal degradation of the low surface energy reagent PDMS (339 °C), causing the film to lose its superhydrophobicity.^51^ These results suggested that ES improved the thermal stability of PDMS and so the durability of the film, which was consistent with the previous study by Wang et al., where they showed that the thermal degradation temperature of pure PDMS can be increased by modifying PDMS with epoxy-based materials.^52^

**Table 4:** WCAs and SAs for ES/0.6PDMS/0.6SiO_2_ and 0.6PDMS/0.6SiO_2_ Films After 5 h of Heating at 300 °C and 400 °C

	Before heating		After heating at 300 °C for 5 h		After heating at 400 °C for 5 h	
	ES film	Film without ES	ES film	Film without ES	ES film	Film without ES
WCA/°	165 ± 3	163 ± 2	164 ± 2	149 ± 1	166 ± 2	118 ± 4
SA/°	2	3	2	10	2	N/A

Figure S7 demonstrates the excellent UV stability of ES/0.6PDMS/0.6SiO_2_ and 0.6PDMS/0.6SiO_2_ films. After 2 weeks of UV exposure, there was no obvious change in the wettability of both films, with WCAs > 160° and SAs < 5°. It is well known that UV irradiation is harmful to materials and can lead to material degradation. Since ES, PDMS, and SiO_2_ NPs are chemically inert and not photoactive, both films showed good UV resistance.

Furthermore, the self-cleaning and stain tests for the ES/0.6PDMS/0.6SiO_2_/360 °C/40 min film were also carried out. As shown in Figure S8, gold glitter was used to represent dirt, which was evenly spread onto the as-prepared SH glass surface. As the water droplets slid off, the glitter was carried away and left a clean path, which was attributed to its excellent self-cleaning feature. For the stain test, distilled water containing methylene blue was used for visualization, and multiple droplets were dropped onto the coating. The water droplets readily rolled off the coating, leaving a dry, clean surface. This self-cleaning ability and nonstaining feature was due to the high surface roughness and low surface energy, which reduced the contact and adhesion between the surface and droplets.

This study has shown that one layer of the hydrophilic ES base coat did not affect the water repellency of the films. Depositing PDMS onto the ES-pretreated glass via AACVD yielded a SH surface with a WCA of 155 ± 2°. The addition of SiO_2_ NPs was able to further increase the roughness, achieving WCAs > 160°. The superhydrophobicity of the ES/PDMS/SiO_2_ film was comparable to that of fluorinated or ER/PDMS/SiO_2_ films reported in the literature (∼162°).^11,19,30,40^ By performing robustness tests on both ES/PDMS/SiO_2_ and PDMS/SiO_2_ films, we proved that ES significantly improved the durability of the films. In this study, the high robustness achieved by ES was comparable to that achieved by ER, a commonly used adhesive in previous reports.^19−21^ In addition, the use of the AACVD technique in our work simplified the fabrication process compared to other approaches, as films with excellent superhydrophobicity can be prepared at a relatively low temperature (360 °C) and in a short time (40 min) without using large amounts of reagents, making them more suitable for large-scale synthesis.

## Conclusions

In this study, robust and translucent superhydrophobic films with excellent self-cleaning abilities were successfully generated by depositing a fluorine-free precursor containing PDMS and silica NPs via AACVD onto the glass substrate pretreated with ES. The optimum film, deposited with 0.6 g of PDMS and 0.6 g of SiO_2_ at 360 °C for 40 min, displayed outstanding superhydrophobicity (WCA = 165 ± 3°, SA = 2°) and semitransparency. More importantly, the robustness tests showed that SH features remained after 400 tape peel cycles, 5 h of immersion in ethanol, diethyl ether, and toluene, 5 h of heating at 300 and 400 °C, and 2 weeks of UV exposure. The film showed enhanced durability compared to the film without an ES underlayer, where the surface became hydrophobic after 50 tape peel cycles and the SH features were unstable to organic solvents and high temperatures. Those coatings benefited from the ES acting as an adhesive for SiO_2_ NPs that increased the roughness and transparency. The low surface energy reagent PDMS also contributed to the roughness and durability. By comparing the robustness of films with and without an ES underlayer, this study demonstrated that ES is a potentially useful binder, thus providing a new and environmentally friendly adhesive for producing well-adhered SH coatings that can be used in various fields, such as self-cleaning windows, rainwear, aircraft, pavements, and windscreens.

## Supporting Information Available

Characterization, robustness, and self-cleaning testing for the ES/PDMS/SiO_2_ and PDMS/SiO_2_ films (PDF)
