# Supplementary material for: Robust, Fluorine-Free Superhydrophobic Films on Glass via Epoxysilane Pretreatment
Source: Langmuir. 2025 Jan 16;41(3):1556–67. doi: 10.1021/acs.langmuir.4c02630 (PMC11780728; doi:10.1021/acs.langmuir.4c02630)
Supplement: Supplementary file 1 — la4c02630_si_001.pdf [file la4c02630_si_001.pdf]

Supporting information

Robust, Fluorine-Free Superhydrophobic Films on Glass via Epoxy silane Pretreatment

Fang Chen[a], Julie Jalila Kalmoni[a], Shuhui Li[a], and Claire J Carmalt[a]*

*Corresponding author

[a]Materials Chemistry Centre, Department of Chemistry, University College London, 20 Gordon Street, London WC1H 0AJ, UK

E-mail: c.j.carmalt@ucl.ac.uk

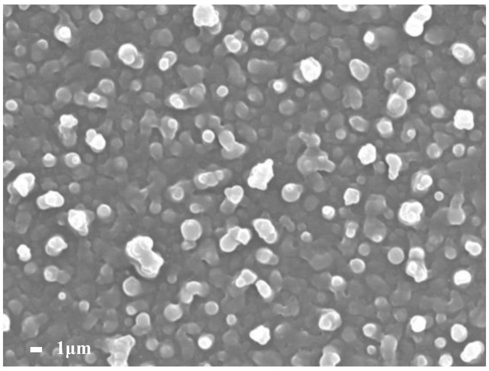

Figure S1. The SEM images of the ES/0.6PDMS film (deposited at 360 °C for 40 min).

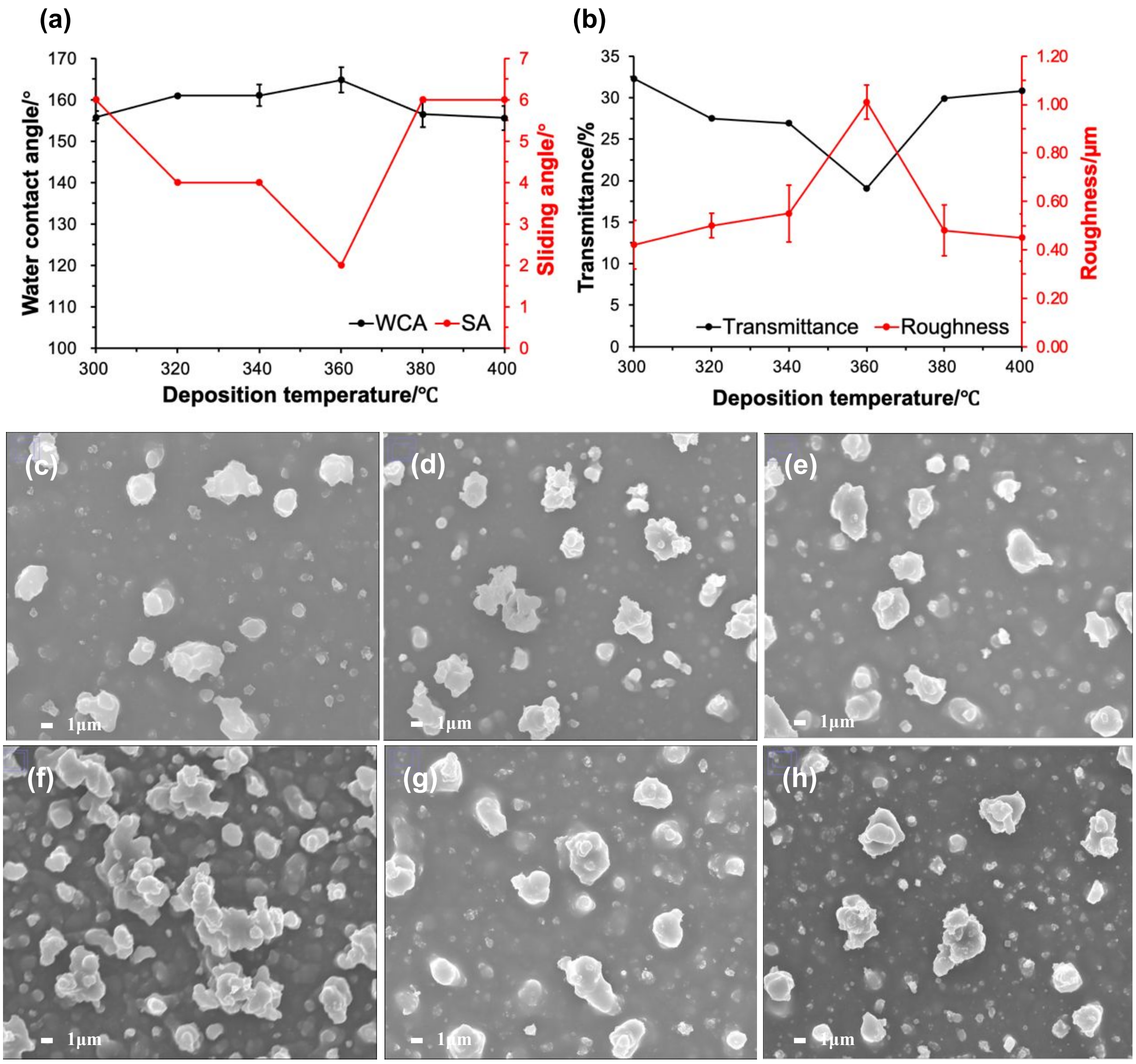

Figure S2. (a) WCAs and SAs (b) transmittance and surface roughness of ES/0.6PDMS/0.6SiO_2 films deposited at 300, 320, 340, 360, 380, and 400 °C. SEM images of ES/0.6PDMS/0.6SiO_2 films deposited at (c) 300, (d) 320, (e) 340, (f) 360, (g) 380, and (h) 400 °C.

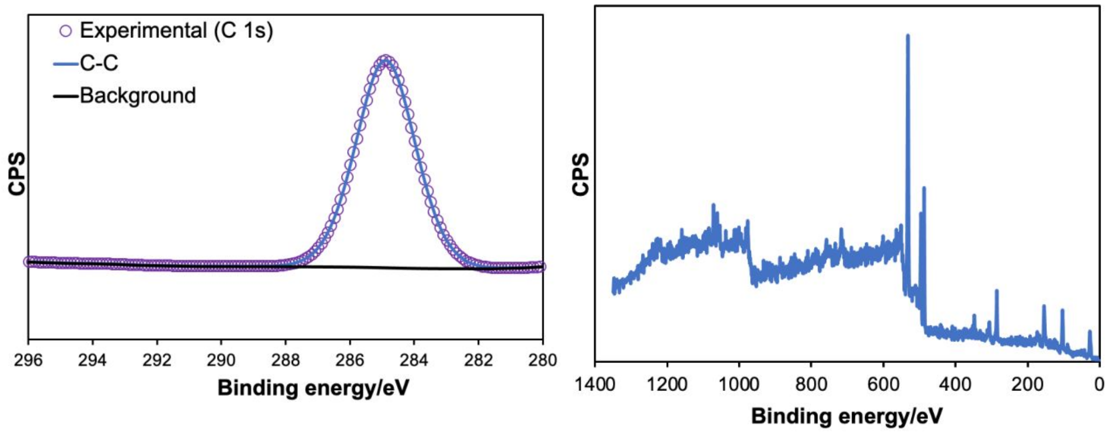

Figure S3. XPS data for the ES/PDMS/SiO_2 film showing the (a) C 1s spectrum and (b) survey spectrum.

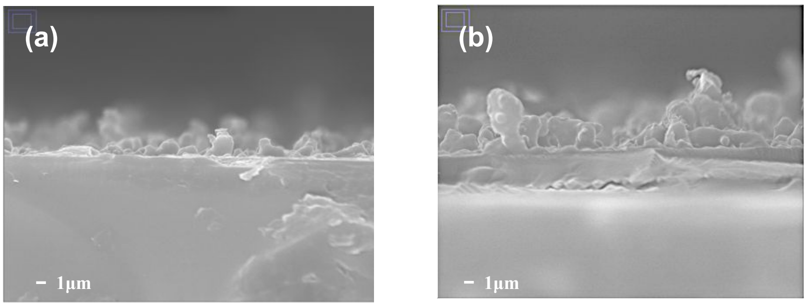

Figure S4. Side-on SEM images of ES/0.6PDMS/0.6SiO_2 films deposited at (a) 15 and (b) 40 min.

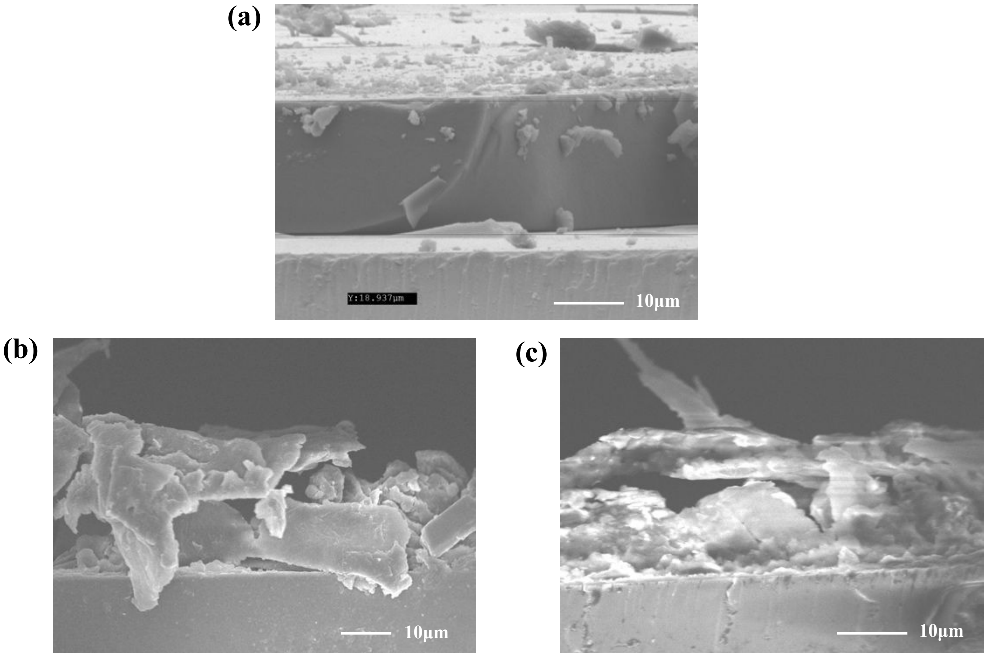

Figure S5. Side-on SEM images of (a) ES, (b) ES/0.6PDMS/0.6SiO_2, and (c) 0.6PDMS/0.6SiO_2 films.

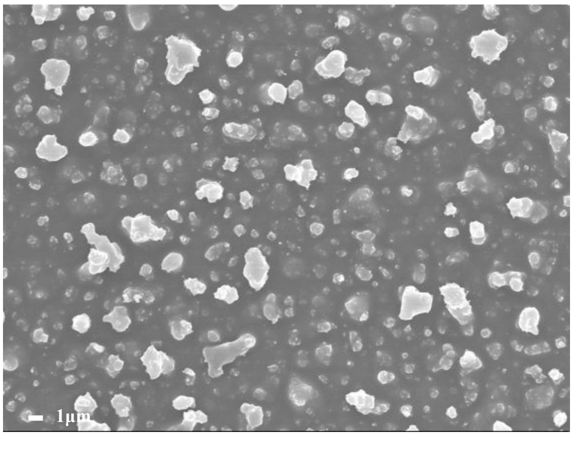

Figure S6. The SEM image of the 0.6PDMS/0.6SiO_2 film (deposited at 360 °C for 40 min).

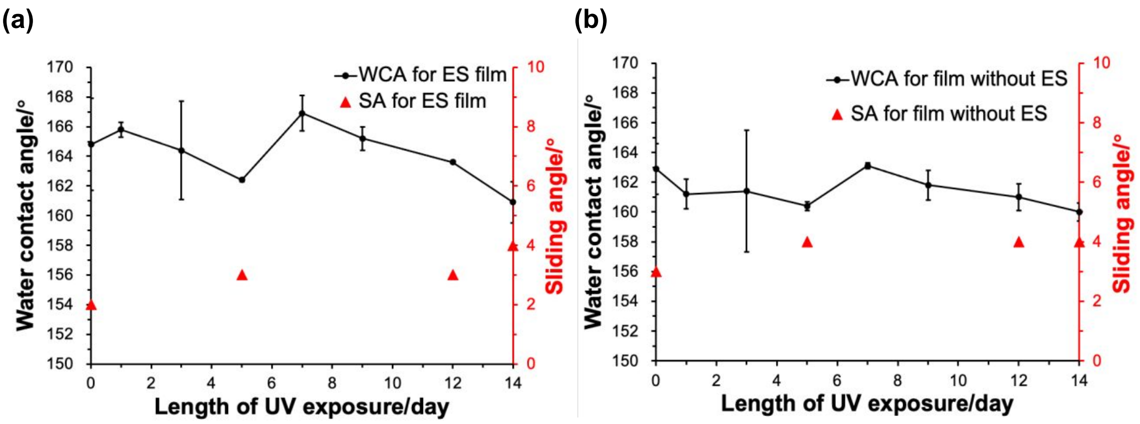

Figure S7. WCAs and SAs for (a) ES/0.6PDMS/0.6SiO_2 and (b) 0.6PDMS/0.6SiO_2 films during 2 weeks of UV exposure.

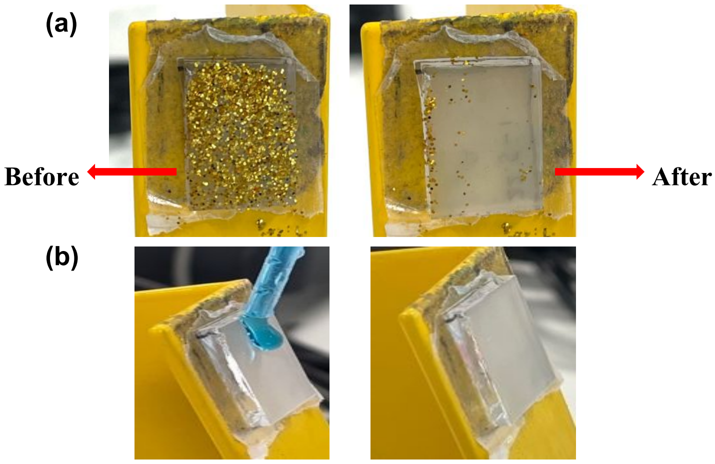

Figure S8. Self-cleaning tests for the ES/0.6PDMS/0.6SiO_2 film using (a) glitter and (b) distilled water containing methylene blue at a tilt angle of 20°.
